# RyR2 and Calcium Release in Heart Failure

**DOI:** 10.3389/fphys.2021.734210

**Published:** 2021-10-08

**Authors:** Jean-Pierre Benitah, Romain Perrier, Jean-Jacques Mercadier, Laetitia Pereira, Ana M. Gómez

**Affiliations:** Signaling and Cardiovascular Pathophysiology—UMR-S 1180, INSERM, Université Paris-Saclay, Châtenay-Malabry, France

**Keywords:** ryanodine receptor, heart failure, calcium, excitation contraction coupling, sinus node, atrial fibrillation

## Abstract

Heart Failure (HF) is defined as the inability of the heart to efficiently pump out enough blood to maintain the body's needs, first at exercise and then also at rest. Alterations in Ca^2+^ handling contributes to the diminished contraction and relaxation of the failing heart. While most Ca^2+^ handling protein expression and/or function has been shown to be altered in many models of experimental HF, in this review, we focus in the sarcoplasmic reticulum (SR) Ca^2+^ release channel, the type 2 ryanodine receptor (RyR2). Various modifications of this channel inducing alterations in its function have been reported. The first was the fact that RyR2 is less responsive to activation by Ca^2+^ entry through the L-Type calcium channel, which is the functional result of an ultrastructural remodeling of the ventricular cardiomyocyte, with fewer and disorganized transverse (T) tubules. HF is associated with an elevated sympathetic tone and in an oxidant environment. In this line, enhanced RyR2 phosphorylation and oxidation have been shown in human and experimental HF. After several controversies, it is now generally accepted that phosphorylation of RyR2 at the Calmodulin Kinase II site (S2814) is involved in both the depressed contractile function and the enhanced arrhythmic susceptibility of the failing heart. Diminished expression of the FK506 binding protein, FKBP12.6, may also contribute. While these alterations have been mostly studied in the left ventricle of HF with reduced ejection fraction, recent studies are looking at HF with preserved ejection fraction. Moreover, alterations in the RyR2 in HF may also contribute to supraventricular defects associated with HF such as sinus node dysfunction and atrial fibrillation.

## Introduction

Heart failure (HF) is one of the major causes of death worldwide. It is characterized by the failure of the cardiac pump to maintain a sufficient blood flow to oxygenize and carry nutrients to the whole body. According to left ventricular systolic function, HF has been divided into two major groups: HF with reduced ejection fraction (HFrEF) and HF with preserved ejection fraction (HFpEF). HFrEF generally occurs after cardiac injury (myocardial infarction) or under chronic stress (hypertension), leading to the alteration of contractile function of the heart. It is now well established that alteration of cardiomyocyte Ca^2+^ homeostasis plays a critical role in the development of the pathology, leading to cardiac remodeling, failure of the cardiac pump, and cardiac arrhythmias.

**Figure 1:**
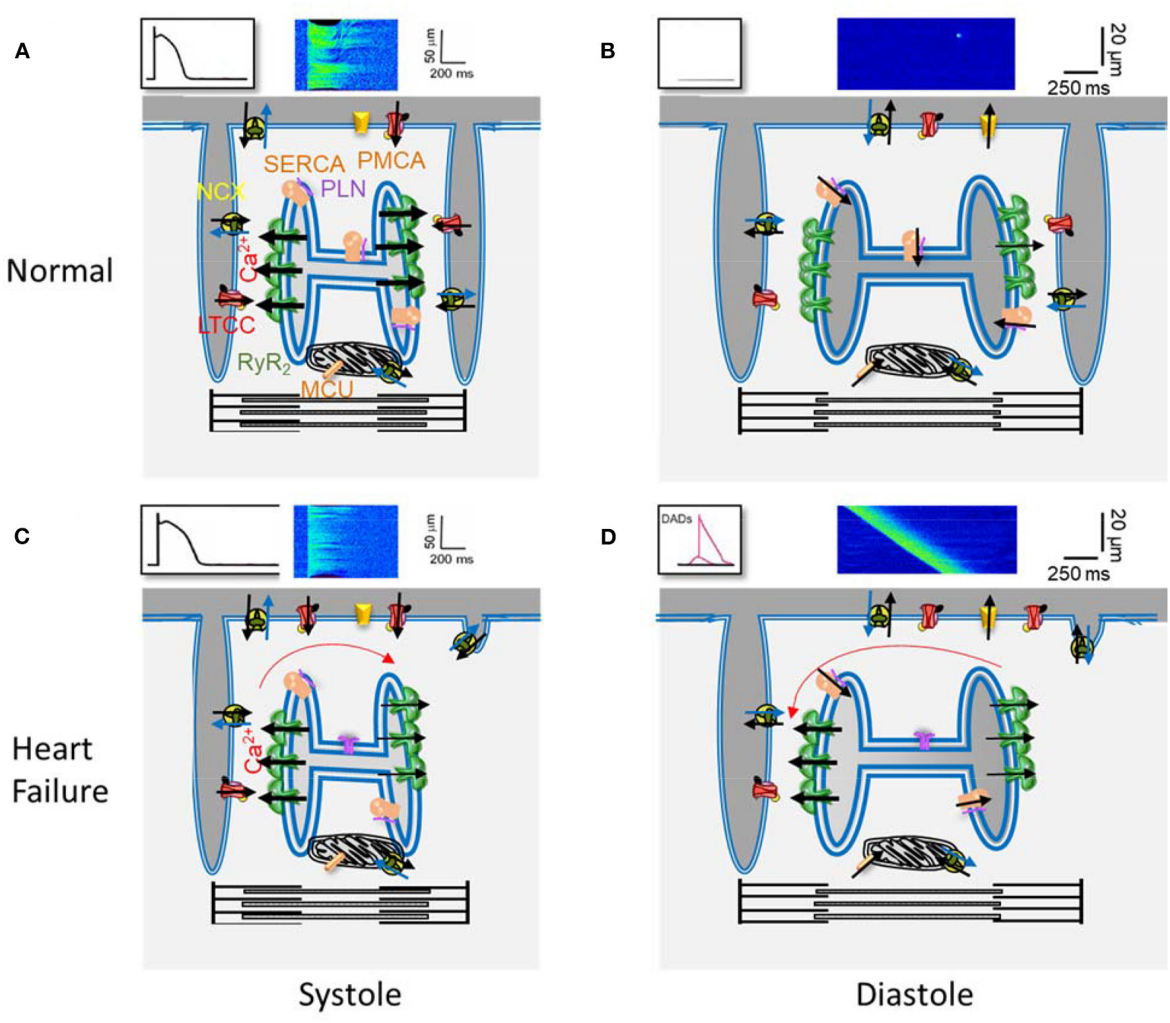
Main elements of Ca^2+^ handling in excitation-contraction coupling in a ventricular cardiomyocyte. **(A)** Scheme of a portion of a ventricular cardiomyocyte showing two T-tubules, with L type Ca^2+^ channels (LTCC) and the Na^+^/Ca^2+^ exchanger (NCX), and the junctional sarcoplasmic reticulum with Ryanodine Receptors (RyR2). Plasmalema and SarcoEndoplasmic Ca^2+^ ATPases (PMCA and SERCA) are also shown, with phospholamban (PLN) which slows SERCA activity, as well as the mitochondrial Ca^2+^ uniporter (MCU). The black arrows indicate Ca^2+^ movements during the action potential (drawing on top left), producing the [Ca^2+^]_i_ transient (confocal line scan image on top). **(B)** The same elements during rest. The LTCC are inactive, and the RyR2 closed, with small leak. **(C)** and **(D)** The same as in **(A)** and **(B)** but in heart failure: the density of T-tubules is decreased as well as SERCA expression; the RyR2 are more active.

Ca^2+^ plays a key role in cardiomyocyte contraction. In each heartbeat, the membrane depolarization during an action potential (AP) activates L-type Ca^2+^ channels (LTCC), which are located in the sarcolemma and are more concentrated at the transverse (T) tubules (Figure 1). The local increase in [Ca^2+^]_i_ in the dyads that follows activates the Ca^2+^ release channels, the ryanodine receptors (RyR2) located in the neighborhood, on the junctional sarcoplasmic reticulum (SR), resulting in the coordinated and global increase in the cell [Ca^2+^]_i_, by the Ca^2+^-induced Ca^2+^-release (CICR) mechanism, activating contractile myofibrils. Relaxation happens when the cytosolic [Ca^2+^]_i_ decreases, mainly by Ca^2+^ re-uptake into the SR through the SarcoEndoplasmic Reticulum Ca^2+^ ATPase (SERCA) and extrusion out of the cell through the Na^+^/Ca^2+^ exchanger (NCX). Other systems, such as the plasmalemmal Ca^2+^ pump and the mitochondrial Ca^2+^ uniporter, play a minor role in cytosolic Ca^2+^ removal. The RyR2 are not so sensitive to Ca^2+^ so their activation probability depends on their proximity to the LTCCs, which determines the local Ca^2+^ concentration (Stern, 1992).

During diastole, some Ca^2+^ leaks outside the SR through the RyR2s, which have very low open probability at low cytosolic [Ca^2+^]_i_, but are also sensitive to luminal Ca^2+^, what is high during diastole. The opening of a RyR2 cluster can be viewed visualized with a confocal microscope and adequate fluorescence dyes, as rapid (~10 ms), brief (~30 ms), and local (~1.5 μm) elevations in cytosolic Ca^2+^, named Ca^2+^ sparks (Cheng and Lederer, 2008). Ca^2+^ sparks analyses provide insight into RyR2 function in *situ*, while the incorporation of RyR2 into lipid bilayers provides direct information on the channel function in a non-cellular context. During excitation-contraction coupling (ECC), Ca^2+^ release units (CRU) formed by a cluster of RyR2s are activated by the LTCCs located in the T tubules in front of them, which open during the AP. The spatial and temporal summation of these coordinated Ca^2+^ sparks results in the whole [Ca^2+^]_i_ transient. The efficacy of the LTCCs to activate CRUs has been referenced as CICR gain, and it has been studied in HF combining patch-clamp to measure L-type Ca^2+^ current (I_Ca_) and confocal microscopy to visualize changes in ≪Ca^2+*i*^.

In HF, there are alterations in contraction and relaxation, which may be related to alterations in cardiomyocyte Ca^2+^ cycling. Alterations have been reported in several cardiomyocyte Ca^2+^ cycling elements, such as SERCA, ratio phosphlamban (PLN)/SERCA, and single LTCCs, during both experimental and human HF. In this review, we will focus on alterations in the RyR2 function on left ventricular HF, although alterations in Ca^2+^ handling have also been found in right ventricular failure (Hautefort et al., 2019).

## Gain of CICR

The first study of Ca^2+^ sparks in HF showed fewer Ca^2+^ sparks for a given amount of Ca^2+^ entry through LTCCs (I_Ca_). This decrease in CICR gain was observed in experimental HF, due to chronic hypertension (Gomez et al., 1997) and after myocardial infarction (Gomez et al., 2001), and was reminiscent of pioneer work by Beuckelmann in human ventricular myocytes from explanted failing hearts, where they found a decrease in the global [Ca^2+^]_i_ transient but normal I_Ca_ density (Beuckelmann et al., 1992). One explanation of this decrease in CICR gain was that there could be an ultrastructural disarrangement, which physically increased the distance between CRU and LTCC. In fact, while I_Ca_ density is generally maintained in HF, higher activity of at the single channel level has been reported (Schroder et al., 1998). This finding suggests fewer LTCC in the T-tubules participating in CICR or a decrease in the T-tubules density, as observed in HF (Wagner et al., 2012; Guo et al., 2013; Jones et al., 2018). But this “uncoupling” of the ECC was also suggested in larger mammals, in which the T-tubular network is less developed. Following myocardial infarction, cardiomyocytes from pigs with HF had relatively more uncoupled RyR2s, which could underlie the decreased gain of CICR (Dries et al., 2018).

Besides this decrease in CICR gain, alterations in the RyR2 itself have been reported in different models of HF.

## RyR2 Post Translational Modifications

During HF progression, the sympathetic nervous system and renin-angiotensin-aldosterone system are activated to compensate for the reduced contractile function of the heart and thus maintain a sufficient blood flow (Lymperopoulos et al., 2013). In this early stage of cardiac remodeling, ECC is enhanced, with an increased [Ca^2+^]_i_ transient amplitude and contraction, together with a faster relaxation (Ohkusa et al., 1997). However, chronic activation of both systems has deleterious effects on cardiomyocytes and is responsible for the decompensation of cardiac function. This decrease in cardiac contraction is associated with an alteration of ECC (Gomez et al., 1997, 2001), the [Ca^2+^]_i_ transient amplitude is decreased and its duration is increased. Besides, or on top of the ultrastructural remodeling mentioned above, the reduction in [Ca^2+^]_i_ transient amplitude can be explained by a reduction of Ca^2+^ stored in the sarcoplasmic reticulum (Piacentino et al., 2003; Lehnart et al., 2009). While the first analyses explained this decrease in SR Ca^2+^ load by a lower SERCA expression and function and/or a higher expression of its natural inhibitor PLN, or a decrease in its phosphorylation (Kranias and Hajjar, 2012), some authors attributed it to RyR2 hyperactivity, which enhances diastolic SR Ca^2+^ leak. Indeed, an increase of the spontaneous Ca^2+^ leak through RyR2 has been observed in HF (Fischer et al., 2014; Ho et al., 2014; Grimm et al., 2015; Fu et al., 2016; Uchinoumi et al., 2016; Walweel et al., 2017; Dries et al., 2018). This SR Ca^2+^ leak, together with the reduced SERCA activity, contributes to a decrease in SR Ca^2+^ stores and an increase of the propensity of Ca^2+^ waves (Cheng et al., 1996; Venetucci et al., 2008; Curran et al., 2010; Dries et al., 2018). This contrasted with a significant decrease in the Ca^2+^ spark frequency reported in isolated cardiomyocytes from patients with terminal HF compared with non-failing individuals (Lindner et al., 2002). As spontaneous Ca^2+^ sparks depend on the amount of SR Ca^2+^ stored and [ATP], which are both reduced in cardiomyocytes from failing hearts, these can mask the increased activity of RyR2, whereas this higher RyR2 activity can still favor Ca^2+^ waves once the Ca^2+^ spark is produced, as the neighboring RyR2 will be more sensitive (Ruiz-Hurtado et al., 2015). Extrusion of Ca^2+^ constituting the Ca^2+^ waves during diastole *via* the NCX can generate delays after depolarization and trigger new action potentials that can propagate and induce arrhythmias (Pogwizd et al., 2001; Venetucci et al., 2008; Belevych et al., 2011). All these modifications of Ca^2+^ release have not been associated with alterations of RyR2 expression during HF (Hasenfuss and Pieske, 2002), but with post-translational modifications such as phosphorylation and oxidation (Houser, 2014). We should also keep in mind that while post-translational alterations in RyR2 tends to increase its activity, other alterations in Ca^2+^-handling proteins (such as SERCA reduction) and metabolic changes (that may curse with lower ATP levels) tend to reduce its activity (Ruiz-Hurtado et al., 2015).

In 2000, it was shown that hyperphosphorylation of RyR2 by PKA is responsible for the leaky RyR2 in failing hearts in humans. The authors claimed that PKA-mediated phosphorylation dissociates the FK506 binding protein (FKBP12.6) from the RyR2 leading to an increase in the channel open probability (Marx et al., 2000). FKBP12.6 is known to stabilize the RyR2 in a closed state and avoid aberrant Ca^2+^ leak (Brillantes et al., 1994; Gellen et al., 2008), but its removal may not affect RyR2 open probability (Xiao et al., 2007). In the early 90s, serine 2808 (S2808) was identified as the unique phosphorylation site of RyR2 (Witcher et al., 1991) and appeared to be a prominent target for PKA. In following publications, Marks' group highlighted the role of S2808 phosphorylation in the destabilization of the complex RyR2-FKBP12.6. They developed RyR2-S2808A knock-in mice, which prevent phosphorylation at this site. They showed that these mice are protected against RyR2-FKBP12.6 dissociation and leaky RyR2 in response to catecholamine stimulation (Shan et al., 2010a,b). Moreover, RyR2-S2808A knock-in mice seem to have a better cardiac function after myocardial infarction, which has been related again to the stabilization of the RyR2-FKBP12.6 complex in the absence of PKA phosphorylation (Wehrens et al., 2006). They also confirmed this major role of S2808 using RyR2-S2808D knock-in mice, which mimicked hyperphosphorylation at that site and had a reduced association of FKBP12.6 to RyR2 (Shan et al., 2010a,b). However, other groups have not been able to confirm this major role of S2808 phosphorylation in the progression of HF (Jiang et al., 2002). Valdivia and Houser's groups also developed RyR2-S2808A knock-in mice. In these mice, response to β-adrenergic stimulation on [Ca^2+^]_i_ transient and ECC gain (MacDonnell et al., 2008) and progression of HF after myocardial infarction (Zhang et al., 2012) or aortic banding (Benkusky et al., 2007) were not affected no matter the genetic background of the mice (Alvarado et al., 2017). Moreover, it has also been shown that FKBP12.6 binding to RyR2 is not modified by acute β-adrenergic stimulation on wild-type mice or FKBP12.6 overexpressing mice in the heart (Gellen et al., 2008). Recently, evidences that S2808 is not the only target for RyR2 phosphorylation by PKA have been put forward; S2030 seems to be a potent target of PKA (Potenza et al., 2019) and can be involved in the progression of HF (Xiao et al., 2006; Benkusky et al., 2007). Taken together, these studies are in contradiction with Marks' group publications, and the controversy continues through the years (Bers, 2012; Alvarado and Valdivia, 2020; Dridi et al., 2020a,b). However, independently on RyR2 phosphorylation status, FKBP12.6 has been more consistently found decreased in HF (Ono et al., 2000; Gomez et al., 2009), which can by itself promote diastolic Ca^2+^ leak.

The Ca^2+^/calmodulin-dependent protein kinase II (CaMKII) phosphorylation of S2814 (2815) has also been highlighted for its role in the increase in SR Ca^2+^ leak through RyR2 in HF. CaMKII is activated by catecholamine and by oxidation, which is high during HF (Erickson et al., 2008). It has been shown that in failing rabbit hearts, CaMKII phosphorylation of RyR2 decreases SR load and increases Ca^2+^ leak by increasing RyR2 open probability (Ai et al., 2005). In these rabbits, inhibition of CaMKII, but not of PKA, reduced SR Ca^2+^ leak (Ai et al., 2005). In human heart failure due to ischemic or dilated cardiomyopathy, CaMKII expression has been shown to increase, contributing to augmented SR Ca^2+^ leak (Fischer et al., 2014). Rats subjected to myocardial infarction treated with exendin-4, which reduced CaMKII activity, showed a decreased SR Ca^2+^ leak (Chen et al., 2020). This major effect of CaMKII in the development of HF has been confirmed in mice lacking CaMKII. After transverse aortic constriction, these mice were protected against HF progression together with a reduced SR Ca^2+^ leak and RyR2 phosphorylation at S2815 (Ling et al., 2009). Similarly to CaMKII Knock out mice, RyR2-S2814A knock-in mice were protected against abnormal SR Ca^2+^ leak and HF after transverse aortic constriction (TAC) (van Oort et al., 2010) but surprisingly not after myocardial infarction (MI) (Respress et al., 2012). To explain how CaMKII increases SR Ca^2+^ leak, Uchinoumi et al. hypothesize that phosphorylation of S2814 reduces CaM affinity for the RyR2, leading to a RyR2 conformational change and leakiness of the channel (Uchinoumi et al., 2016). Supporting this hypothesis, Dantrolene, which restores RyR2 CaM affinity, suppressed SR Ca^2+^ leak in RyR2 S2814D knock-in mice (Uchinoumi et al., 2016).

Among the 90 cysteines present in each of the four subunits of RyR2, 21 are in a free thiol state and accessible for redox modifications during oxidative stress (Xu et al., 1998). Oxidative stress is associated with the development of several pathologies, and excessive production of reactive oxygen species has deleterious effects on protein function and cell viability. To counter these deleterious effects, cells have their own specific antioxidants machinery, including superoxide dismutase, catalase, and glutathione peroxidase, and non-specific antioxidants reduced glutathione, which is ubiquitous and the most important antioxidant system in cardiac cells (Nikolaienko et al., 2018).

During HF ROS production is chronically increased due to the uncoupling of the mitochondrial electron transport chain, the increase in energy demand, the switch from fatty acids to glucose as an energy substrate in cardiomyocytes (Mak and Newton, 2001; Ventura-Clapier et al., 2004), upregulation of nitric oxide synthase (NOS), xanthine oxidase and NADPH oxidase (NOX), and decreased reduced glutathione (Zima and Mazurek, 2016; Nikolaienko et al., 2018). ROS are also known to activate SR Ca^2+^ leak (Terentyev et al., 2008) by increasing spontaneous Ca^2+^ sparks frequency (Yan et al., 2008; Prosser et al., 2011) and Ca^2+^ waves (Bovo et al., 2012). Indeed, part of the beneficial effect of carvedilol on HF has been attributed to its antioxidant action on RyR2 (Mochizuki et al., 2007). ROS oxidation of RyR2 during HF induces the dissociation of FKBP12.6 leading to the aberrant Ca^2+^ release (Shan et al., 2010a), although other groups showed that RyR2 oxidation induces CaM dissociation without changing FKBP12.6 binding (Ono et al., 2010; Oda et al., 2015). Increased NOX activity induced by tachycardia led to an increase in RyR2 *S*-glutathionylation associated with an increased SR Ca^2+^ leak (Sanchez et al., 2005), which has been suggested to be cardio protective during preconditioning with exercise. However, the role of NOX during HF is not well-documented. The increased activity of NOX during end-stage HF (Heymes et al., 2003) can participate in the increase in diastolic SR Ca^2+^ leak and SR Ca^2+^ depletion. Even if hypernitrosylation of RyR2 is supposed to increase Ca^2+^ leak, decreased *S*-nitrosylation has been associated with increased SR Ca^2+^ leak during HF (Gonzalez et al., 2007; 2010). In fact, it has been proposed that hyponitrosylation during HF favors the oxidation of RyR2 by ROS, leading to this aberrant Ca^2+^ release. In these studies, inhibition of xanthine oxidase decreased ROS production restoring *S*-nitrosylation and cardiac function (Gonzalez et al., 2010). Evidence of the cardio protective effect of RyR2 *S*-nitrosylation has been also highlighted in a mouse model where NOS1 overexpression prevents cardiac dysfunction and delays HF in response to pressure overload (Loyer et al., 2008). On a canine model of HF, it has been suggested that the disulfide oxidation is the predominant form of redox-sensitive modulation of RyR2 compared to *S*-nitrosylation and *S*-glutathionylation to influence the RyR2-mediated leak. On a rabbit model of HF, increased SR Ca^2+^ leak (Mazurek et al., 2014) and Ca^2+^ waves (Bovo et al., 2015) could be attributed to an increase in RyR2 intersubunit disulfide cross-linking (Mazurek et al., 2014; Bovo et al., 2015, 2018).

By accessing data from different laboratories, we can gather that the RyR2 itself may be more active in HF due to several post-translational modifications such as oxidation, enhanced phosphorylation at the CaMKII site, as well as a depressed FKBP12.6 expression/binding to RyR2.

Figure 1 summarizes the key elements in Ca^2+^ handling in ventricular cardiomyocytes, as well as a representation of some of the reported findings in those elements, in different models of HF, which contribute to depressed contraction and pro-arrhythmogenic DADs production. The failing cell has a disruption in the T-tubular network, and a decreased SERCA expression (or augmented ratio PLN/SERCA), which together with more active RyR2 due to post-translational modifications, resulting in a drop of Ca^2+^ stored in the SR. As a result, and despite normal I_Ca_ density and prolonged AP, the triggered [Ca^2+^]_i_ transient is of lower amplitude, contributing to diminished cell contraction. During diastole, the higher RyR2 activity favors the probability of producing Ca^2+^ waves, generating an inward current through the NCX, when extruding Ca^2+^, which produces DADs. When DADs reach the threshold, a triggered AP is generated, which may initiate arrhythmias.

## HF With Preserved Ejection Fraction (HFpEF)

HF with preserved ejection fraction (HFpEF) has been identified as a subtype of HF in addition to HFrEF. HFpEF is more frequently found in older patients with comorbidities such as hypertension, obesity, diabetes, chronic kidney diseases (Redfield et al., 2003; Bhatia et al., 2006; Owan et al., 2006). HFpEF patients present normal left ventricular systolic function with impaired left ventricular relaxation and filling. Although HFpEF represents approximately 50% of HF patients (Dunlay et al., 2017), little is known about their specific mechanisms, in comparison to HFrEF, notably regarding RyR2 and Ca^2+^ release. A lack of knowledge is probably related to the difficulty in generating accurate experimental models or the diversity of associated comorbidities. As described previously, in HFrEF, cardiac systolic and diastolic changes are linked to Ca^2+^ mishandling. In HFpEF, Ca^2+^ alterations are not as clearly defined and seem to differ depending on the associated comorbidity. In a rat model in HFpEF with chronic pressure overload, a significant increase in diastolic SR Ca^2+^ release through RyR2s leading, as in HFrEF, to higher diastolic [Ca^2+^] and irregular [Ca^2+^]_i_ transients during pacing has been observed (Rouhana et al., 2019). This SR Ca^2+^ leak has been associated with an increase in PLN/SERCA ratio responsible for a delayed [Ca^2+^]_i_ transient decay and diastolic dysfunction, also seen in HFpEF with chronic kidney disease (Primessnig et al., 2016) and in human HF with EF > 45% (Hohendanner et al., 2013; Ljubojevic et al., 2014). Unlike HFrEF, a SR Ca^2+^ leak in HFpEF depends on PKA-dependent phosphorylation of RyR2 at S2808 (Durland, 2021) rather than CaMKII-dependent phosphorylation, alteration of FKBP12.6/RyR2 ratio, or RyR2s *S*-nitrosylation (Adeniran et al., 2015; Frisk et al., 2021). However, phosphorylation of RyR2 at S2808 was not significantly changed in Dahl salt-sensitive rats, with HFpEF, presenting both hypertension and insulin-resistance (Kilfoil et al., 2020). This discrepancy could be explained by insulin-resistance comorbidity not present in Durland's model (Durland, 2021). Although in HFrEF an increase in SR Ca^2+^ leak leads to SR Ca^2+^ depletion and lower [Ca^2+^]_i_ transient, in HFpEF, diastolic SR Ca^2+^ release does not induce SR Ca^2+^ load depletion or defective ECC. In fact, in HFpEF models, systolic Ca^2+^ release by the RyR2 is increased, probably as a compensatory mechanism to overcome the increased left ventricular stiffness associated with HFpEF (Selby et al., 2011; Adeniran et al., 2015; Durland, 2021). This enhanced SR Ca^2+^ release could be attributed to a more effective RyR2s recruitment by Ca^2+^ during the Ca^2+^ influx through the LTCC (Kilfoil et al., 2020) as well as an absence of T-tubules alteration (Durland, 2021). Indeed, in a Dahl-sensitive rat model of HFpEF (Kilfoil et al., 2020), a higher couplon recruitment improved ECC by increasing Ca^2+^ release synchronicity and lowering Ca^2+^ release latency. This should allow the heart to maintain an effective contraction besides higher ventricular wall stiffness. Moreover, T-tubule disorganization might also affect Ca^2+^ release effectiveness. However, the integrity of T-tubule structure in HFpEF depends on the comorbidities. Indeed, while T-tubule density is unchanged in Dahl salt-sensitive rats, in obese and diabetic Zucker rats or diabetic HFpEF patients, their density is increased in the ischemic model (post-MI with preserved EF but reduced E/A) (Frisk et al., 2021). This etiology-dependence is also found in proteins regulating SR Ca^2+^ release such as SERCA and NCX. In diabetic HFpEF, NCX expression and activity of SERCA decrease, which is not the case in ischemic or hypertensive disease models (Frisk et al., 2021). In type 1 diabetes, with subclinical diastolic dysfunction and normal systolic function, diastolic SR Ca^2+^ release and amplitude (measured as Ca^2+^ sparks frequency) decrease and is associated with a drop of SR Ca^2+^ load and [Ca^2+^]_i_ transient without affecting systolic function (Lagadic-Gossmann et al., 1996; Lacombe et al., 2007; Hamouda et al., 2015). In these diabetic models, SR Ca^2+^ uptake by the SERCA pump appears as the main responsible for the decrease in SR Ca^2+^ release with, in some models, an alteration of the SERCA2/PLN ratio (Miranda-Silva et al., 2020). It is deduced from the literature that the alterations in the RyR2s and Ca^2+^ release diverge between HFpEF depending on their diabetic or non-diabetic etiology, with a clear implication of SERCA alteration in the Ca^2+^ homeostasis underlying the diastolic function in diabetes.

Interestingly, in myocardial strips from patients with hypertrophy associated with hypertension and coronary artery disease, but normal ejection fraction (LVEF ≥ 50%), diastolic dysfunction is associated with an increase in SR Ca^2+^ load at high pacing rates (Selby et al., 2011). However, the impact of cellular changes in Ca^2+^ handling does not always transduce into *in vivo* alterations. Indeed, in isolated myocardial strips from animal models of hypertrophy induced by aortic banding, even though cellular Ca^2+^ extrusion was increased due to higher NCX and SERCA activities, *in vivo* relaxation remained slower (Roe et al., 2017). Similarly, exacerbated cellular Ca^2+^ handling has been described in a rat strain with cardiac hypertrophy despite the lack of hypertension, where the increased Ca^2+^ influx through the LTCCs seems to underlie the increased [Ca^2+^]_i_ transient amplitude and myocyte shortening. In this model, an increase in RyR2 phosphorylation by CaMKII leads to higher susceptibly to *in vivo* arrhythmia and spontaneous SR Ca^2+^ release although RyR2 expression was decreased (Curl et al., 2018).

## Alteration of RyR2 in HF Supraventricular Function

### RyR2 and Sinus Node Dysfunction

In addition to its role in governing cardiac contraction, the RyR2-mediated Ca^2+^ release of the pacemaker cardiomyocytes of the heart, located at the sinus node (SAN), contributes to heart automaticity. This observation was initiated in the late 80s by the finding that ryanodine slows down the final phase of diastolic depolarization, resulting in a significant increase in cellular pacemaker cycle length in cats (Rubenstein and Lipsius, 1989). Since then, and after intense debates, it is now of general agreement that spontaneous cyclical local Ca^2+^ releases named late Ca^2+^ release (LCR), are responsible for a net depolarizing current mediated by the NCX. This process drives pacemaker depolarization, which is referred to as the “Ca^2+^ clock,” jointly with the “membrane or voltage clock” mediated by cyclic activation and deactivation of voltage-sensitive membrane ion channels [see for review (Carmeliet, 2019; Kohajda et al., 2020)]. This has been especially emphasized by the fact that RyR2 mutations associated with catecholaminergic polymorphic ventricular tachycardia (CPVT) induced SAN dysfunction (Neco et al., 2012; Wang et al., 2017).

Heart rate (HR) is an independent risk factor of all-cause mortality, cardiovascular mortality, and hospitalization for HF (Fox et al., 2007; Verrier and Tan, 2009). It has been long recognized that HF is associated with dysfunction of the SAN. Whereas, HR is commonly increased due to an excess of sympathetic activity and parasympathetic withdrawal, the intrinsic sinus rhythm in absence of autonomic nerve activity is depressed in HF. In the late 40s, a slower HR in isolated failing hearts was shown (Wollenberger, 1947; Wiggers, 1949). In animal models, and more importantly in humans with HF, the intrinsic HR is decreased with a reduction in SAN reserve and abnormal propagation of APs from the SAN, together with a caudal shift of the leading pacemaker site and fibrosis (Jose and Taylor, 1969; Jose and Collison, 1970; Vatner et al., 1974; Opthof et al., 2000; Verkerk et al., 2003; Sanders et al., 2004; Zicha et al., 2005; Packer et al., 2009; Swaminathan et al., 2011; Yanni et al., 2011). It has been suggested that this anatomic, structural, and functional SAN remodeling in HF might be an adaptive, protective response to improve cardiac oxygen supply to demand ratio (Mulder and Thuillez, 2006) and to prevent triggered arrhythmias enhanced by rapid HR (Opthof et al., 2000). However, reduced SAN automaticity, which favors the induction of early after-depolarization-triggered arrhythmias (Nuss et al., 1999), might translate into bradyarrhythmias or tachycardia-bradycardia syndrome (Mangrum and DiMarco, 2000). Indeed, bradyarrhythmias account for up to half of the deaths in HF (Luu et al., 1989; Stevenson et al., 1993; Uretsky and Sheahan, 1997; Faggiano et al., 2001; Packer et al., 2009; Bloch Thomsen et al., 2010; Gang et al., 2010; Glukhov et al., 2013; Lou et al., 2014). Moreover, in association with autonomic dysfunction (Colucci et al., 1989; Bristow et al., 1990; Samejima et al., 2003; Messias et al., 2016), slowing of the intrinsic HR by SAN remodeling might limit HR modulation to exercise (Sanders et al., 2004), the chronotropic incompetence observed during the HF process (Weber et al., 1982; Higginbotham et al., 1983; Brubaker et al., 2006; Brubaker and Kitzman, 2011; Benes et al., 2013).

To date, analysis of the Ca^2+^ clock in SAN dysfunction in HF has received little attention. In a canine model of HF induced by rapid pacing and using optical mapping, SAN bradycardia is associated with suppression of LCR together with the unresponsiveness of Ca^2+^ clock to isoproterenol and caffeine stimulation (Shinohara et al., 2010). In a similar rabbit HF model, alterations of SAN electro-pharmacological responses have been related to lower expression of RyR2, as well as inhibition of SERCA reuptake due to altered phosphorylation of PLN (Chang et al., 2017). By contrast, RyR2 expression, along with other Ca^2+^ handling proteins, is increased in the SAN but not in atrial tissue in a HF rat model (Yanni et al., 2011). The authors suggested that conjoint upregulation of RyR3 and calsequestrin 2 might however inhibit Ca^2+^ release resulting in slow automaticity. More recently, the same group (Yanni et al., 2020) reported that pressure overload-induced HF in mice did not change SAN mRNA for various components of the “Ca^2+^ clock,” including RyR2, SERCA, calsequestrin, and NCX. This is consistent with a study in isolated SAN cells from rabbit HF model of pressure and volume overload, which concluded that SAN Ca^2+^ cycling properties are conserved despite bradycardic effects (Verkerk et al., 2015). However, the latest study did not take into account the presence of hierarchical pacemaker clustering within the SAN that might be modified in HF (Sanders et al., 2004; Lang and Glukhov, 2021). Preliminary data from our group, using TAC-induced HF in the mouse indicate impairment of “Ca^2+^ clock,” characterized by slower spontaneous [Ca^2+^]_i_ transients, as well as less frequent and smaller Ca^2+^ sparks, supported by a mechanism including depression in the CaMKII signaling pathway (Xue et al., 2020). Taking into account that only a few (and somewhat controversial) studies have evaluated SAN Ca^2+^ clock function in HF to date, further studies are still clearly needed.

### RyR2 and Atrial Fibrillation

Atrial fibrillation (AF) is a frequent complication of HF, and there is a reciprocal relationship between them. HF favors the development of AF (see below), and AF, because of its hemodynamic deleterious consequences, aggravates and may even generate HF. AF is defined as a rapid and disorganized electrical and mechanical activity of the atria, resulting in the loss of the synchronous contraction of atrial myocytes and myocardium normally occurring at end-diastole. The rapid anarchic electrical activity of the atria (around 300/min) is transmitted to the ventricle through the atrio-ventricular (AV) node, which plays the role of a filter, resulting in an irregular and nevertheless often rapid ventricular contraction rate. This has two deleterious consequences: first, the rapid irregular ventricular rate, together with the loss of atrial contraction, is responsible for a deterioration of ventricular filling, itself resulting in decreased cardiac output; second, the loss of atrial contraction favors blood stasis in the atria and especially the auricles, with the risk of thrombus formation and its migration into a peripheral artery.

As with all cardiac arrhythmias, AF necessitates the coexistence of a substrate (functional or anatomic reentry circuits, and tissue remodeling) and a trigger (increased automaticity or triggered activities), both modulated by alterations of the autonomous nervous system tone (increase in the sympathetic tone in the case of HF) or other humoral alterations such as hypokalemia, hypoxia, or acidosis (Coumel, 1993, 1996). It is important to note that HF can not only participate in but even create these three conditions.

Even if, sometimes, it may appear somewhat artificial, one may distinguish two radically different forms of AF, at least for the sake of clarity. The pathophysiology of HF-induced AF is somewhat different and much less documented than that of the “loan AF” occurring in a presumed normal heart. In the latter, AF originates from the wall of the pulmonary veins (Haissaguerre et al., 1989), starts as rare episodes of short duration (paroxysmal AF) that become more and more frequent and of longer duration with time to then finally become persistent, i.e., unable to end spontaneously to return to sinus rhythm. In this pathophysiological scheme, the recurrence of AF episodes is responsible, with time, for a progressive cellular electrical, Ca^2+^ signaling, and tissue remodeling that favors new AF episodes and prevents the return to sinus rhythm: AF begets AF. A detailed description of this remodeling can be found in excellent recent reviews (Landstrom et al., 2017; Denham et al., 2018).

In HF-induced AF, the pathophysiological process starts from the increased ventricular filling pressure that creates a chronic mechanical overload of the atria, associated with various degrees of neurohumoral activation leading to the atrial remodeling creating a favorable ground (both the substrate and the trigger) for the occurrence of AF. Interestingly, HF generally develops an atrial pro-arrhythmic substrate before AF arises, that may therefore immediately present as persistent (Sisti et al., 2014).

Alterations in Ca^2+^ signaling are instrumental to the two pathophysiological mechanisms, showing several similarities, but also subtle differences. They are involved both in the triggering of AF and in the progression of the atrial substrate that facilitates AF. These alterations are impacted by the atrial nature of cardiomyocytes. Indeed, atrial myocytes have a much less developed T-tubular network than ventricular myocytes with less junctional SR, and RyR2 clusters disconnected from T-tubules (orphan clusters). This favors a more progressive rise and delayed peak of the [Ca^2+^]_i_ transient as compared to ventricular myocytes (Hatem et al., 1997; Caldwell et al., 2014). This also offers a greater opportunity for the development and propagation of Ca^2+^ waves. Thanks to significant technological advances in cell imaging, ECC of mice and human atrial myocytes has been recently clarified. Besides sparse T-tubule invaginations, which is associated with slow Ca^2+^ propagation, a voluminous axial tubular system develops extensive junctions with the SR comprising highly phosphorylated RyR2 clusters responsible, in mouse atrial myocytes, for a Ca^2+^ release approximately two times faster at the center of the cell than at its border, in agreement with the fast contractile activation of atrial myocytes (Brandenburg et al., 2016).

In contrast to the significant number of works exploring the role of Ca^2+^ mishandling in paroxysmal/persistent AF (Landstrom et al., 2017; Denham et al., 2018), far less has been published regarding the effects of HF on atrial Ca^2+^ handling. Studies have been carried out on various types of human atrial samples and two types of experimental models have been developed. The first uses long-lasting rapid ventricular pacing that finally results in increased atrial pressures and atrial dilation associated with various patterns of ventricular remodeling (Yeh et al., 2008; Dibb et al., 2009). More consistent with the actual HF pathophysiology, the second (e.g., MI in rat or rabbit, TAC in the mouse), aims to obtain left ventricular failure with only secondary impact on the left atrium (Boixel et al., 2001; Kettlewell et al., 2013; Brandenburg et al., 2016).

I_Ca_ is generally found to be decreased in atrial myocytes of HF models as well as in dilated atria or other pathological conditions associated with increased susceptibility to AF (Dinanian et al., 2008). In the rat MI model, the decrease in I_Ca_ density was related to a decrease in basal cAMP-dependent regulation of the current (Boixel et al., 2001). I_Ca_ density and the resulting [Ca^2+^]_i_ transient amplitude are also decreased under β-adrenergic stimulation in atrial myocytes isolated from rabbits with MI and increased susceptibility to AF (Kettlewell et al., 2013). In the tachypacing HF model in dogs, [Ca^2+^]_i_ transient amplitude and SR Ca^2+^ load were increased, associated with an increased diastolic Ca^2+^ concentration (Yeh et al., 2008). This was associated with decreased RyR2 and calsequestrin protein expression and increased CaMKII-dependent PLN phosphorylation whereas RyR2 phosphorylation was unchanged. In the mouse TAC model, left atrial hypertrophy is associated with marked proliferation of axial tubules and an increase in phosphorylated RyR2 at S2808, but not S2814, thereby accelerating SR Ca^2+^ release through non-junctional RyR2 cluster sites, despite decreases in RyR2 cluster density and RyR2 protein expression (Brandenburg et al., 2016). In a rabbit model of combined pressure and volume overload, diastolic Ca^2+^ concentration was also increased with [Ca^2+^]_i_ transient of larger amplitude due to enhanced IP3 receptor-induced Ca^2+^ release originating from central non-junctional SR, associated with increased frequency of spontaneous Ca^2+^ waves, increased activity of NCX, and Ca^2+^ wave-triggered action potentials (Hohendanner et al., 2015). Interestingly, reduced RyR2 expression associated with increased sensitivity to ryanodine occurs in the atrioventricular node and participate in the slowing of AV conduction observed with aging (Saeed et al., 2018). This process, probably enhanced in failing hearts, would increase the role of the filter assigned to the AV node and therefore protect the failing ventricles from the deleterious consequences of high beating rates.

As seen in paroxysmal/persistent AF, HF-induced AF is also associated with increased diastolic Ca^2+^ leak despite no change or even decreased RyR2 expression. However, in contrast to the former, in which RyR2 phosphorylation is increased at both the PKA and CaMKII sites, at the stage of persistent AF, RyR2 phosphorylation does not appear to change in HF-induced AF. Therefore, the increased leak is more likely due to the increase in SR Ca^2+^ content, generally observed in models of HF in big mammals (Yeh et al., 2008; Clarke et al., 2015; Aistrup et al., 2017). The increased diastolic Ca^2+^ leak is often associated with increased NCX activity that favors the occurrence of DADs.

Recently, a striated muscle preferentially expressed protein kinase (SPEG) has been shown to play a role in the pathophysiology of paroxysmal/persistent AF (Campbell et al., 2020). Unlike PKA and CaMKII that increase RyR2 activity, SPEG phosphorylation at S2367 reduces RyR2-mediated SR Ca^2+^ release. SPEG protein levels, and RyR2 S2367 phosphorylation are decreased in atrial biopsies from patients with paroxysmal AF and transgenic RyR2-S2367A mice, in which the site cannot be phosphorylated, exhibited an increased susceptibility to pacing-induced AF. Whether such a pathophysiological mechanism exists in HF-induced AF remains to be established.

At last, it should be noted that HF also affects the pulmonary veins by increasing their electrical activity, thereby favoring the incidence of the trigger of paroxysmal/persistent AF in HF-induced AF (Lin et al., 2016).

In summary, the pathophysiology of HF-induced AF is complex and shares many features with paroxysmal/persistent AF, at least regarding alterations in Ca^2+^ signaling. More than in other fields of experimental cardiology, we often observed conflicting results due to differences in the species, experimental models, and disease stage studied. HF-induced AF pathophysiology may differ in humans according to the HF type considered. Indeed, HFpEF, in addition to the hemodynamic overload of the left atrium, may comprise specific pathophysiological mechanisms operating from the disease onset such as those of diabetes, inflammation, oxidation, etc., which may play a direct role simultaneously on the ventricles and atria through a “common atrial and ventricular myopathy” (Packer et al., 2020). Unequivocal information on the pathophysiology of HF-induced AF will be obtained from well-characterized standardized experimental models targeting left ventricular failure with secondary progressive atrial remodeling studied at the various stages of the pathological process.

## Conclusion

In summary, alterations in RyR2 posttranslational modifications, location due to T-tubule remodeling, expression, and binding to accessory proteins have been all found in HF with variable conclusions depending on the experimental models or human etiology. What is clear is that RyR2 alteration contributes to the pathology of HF by participating in the depressed [Ca^2+^]_i_ transient, which is important for depression of cell contraction and in favoring arrhythmogenic Ca^2+^ waves during diastole.

## Author Contributions

All authors contributed to the conception of the manuscript, wrote, edited the manuscript, contributed to the article, and approved the submitted version.

## Funding

This work was funded by Inserm, University Paris-Saclay, and grants from ANR (ANR-15-CE14–0005 and ANR-19-CE14-0031-01) and NIH (2R01HL055438-22).

## Conflict of Interest

The authors declare that the research was conducted in the absence of any commercial or financial relationships that could be construed as a potential conflict of interest.

## Publisher's Note

All claims expressed in this article are solely those of the authors and do not necessarily represent those of their affiliated organizations, or those of the publisher, the editors and the reviewers. Any product that may be evaluated in this article, or claim that may be made by its manufacturer, is not guaranteed or endorsed by the publisher.
